# Emergence of hysteresis loop in social contagions on complex networks

**DOI:** 10.1038/s41598-017-06286-w

**Published:** 2017-07-21

**Authors:** Zhen Su, Wei Wang, Lixiang Li, Jinghua Xiao, H. Eugene Stanley

**Affiliations:** 1 School of Science, Beijing University of Posts and Telecommunications, Beijing, 100876 China; 2 Center for Polymer Studies and Department of Physics, Boston University, Boston, Massachusetts 02215 USA; 3 Web Sciences Center, University of Electronic Science and Technology of China, Chengdu, 610054 China; 4 Big Data Research Center, University of Electronic Science and Technology of China, Chengdu, 610054 China; 5 College of Computer Science and Technology, Chongqing University of Posts and Telecommunications, Chongqing, 400065 China; 6 Information Security Center, State Key Laboratory of Networking and Switching Technology, Beijing University of Posts and Telecommunications, Beijing, 100876 China

## Abstract

Understanding the spreading mechanisms of social contagions in complex network systems has attracted much attention in the physics community. Here we propose a generalized threshold model to describe social contagions. Using extensive numerical simulations and theoretical analyses, we find that a hysteresis loop emerges in the system. Specifically, the steady state of the system is sensitive to the initial conditions of the dynamics of the system. In the steady state, the adoption size increases discontinuously with the transmission probability of information about social contagions, and trial size exhibits a non-monotonic pattern, i.e., it first increases discontinuously then decreases continuously. Finally we study social contagions on heterogeneous networks and find that network topology does not qualitatively affect our results.

## Introduction

Social contagion processes are everywhere range from the spread of health behavior to the diffusion of new products and the spread of innovation^1–5^. Uncovering the spreading mechanisms of different social contagions could help us predict and control contagion dynamics^6–8^. Many successful models for revealing spreading mechanisms have been proposed, and they have found that dynamic processes and critical phenomena are affected by both spreading mechanisms and network topologies^7, 9–11^. Among these spreading mechanisms, social reinforcement plays an important role in social contagions (e.g., the spread of a behavior) but does not exist in biological contagion (e.g., the spread of a disease)^12–17^. In biological contagion, the agents moving out of the susceptible state may move into the latent period before becoming infectious with incorporating the host death^18^. An example of social reinforcement is when a decision made by an individual is affected by the attitudes of neighboring individuals. Watts proposed a threshold model that includes social reinforcement in which an individual becomes active only when a certain fraction of neighboring individuals are in the active state^19^. Using extensive simulations and percolation theory, Watts found that social reinforcement causes the fraction of nodes in the active state versus the average degree to first increase continuously and then decrease discontinuously. Majdandzic and his colleagues found that social reinforcement induces a phase flipping (i.e., hysteresis loop) phenomena that explains some phenomena in real economic networks^20, 21^. Recently Wang *et al*. proposed a non-Markovian behavior spreading model to study the effects of social reinforcement and found that the final adoption size versus the transmission probability can change from discontinuous to continuous^22–24^.

Recently, Iyengar *et al*. studied a real-data about the adoption of a new drug, which also belongs to social contagions^25^. For a period of 17 months they investigated how lunch-time interactions between physicians in three cities—Los Angeles, New York City, and San Francisco—affected their adopting the use of a new drug. They first placed physicians in the susceptible stage (i.e., the physicians have become aware of the drug but have not adopted its use). They found that whether a physician moves into the trial and then into the adoption stage is strongly influenced by the level of social reinforcement provided by peer influence. Their results indicate that the trial stage is existed in the social contagions. Aiming to analyze and control the phenomenon of drug abuse in Italy, Di Clemente *et al*. studied a multi-stage process of drug abuse with considering agents’ ages, death and birth, overdose, budget and social environment^26^. However, we still can not understand the microcosmic spreading mechanisms of social contagions.

In this paper we propose a susceptible-trial-adopted-susceptible (STAS) model to investigate social contagions on complex networks in which an individual in the susceptible state passes through a trial state prior to reaching the adopted state or directly reaches the adopted state. Specifically, an individual in the susceptible state enters the trial state only when their received information about the social contagions (short as ‘information’ in the following text) from neighbors exceeds a given trial threshold, and an individual in the trial state enters the adopted state only when their received information from neighbors exceeds another given adoption threshold. Through extensive simulations and theoretical analyses, we discover the presence of a hysteresis loop. The size of adopted state increases discontinuously as a function of the probability of information transmission, and the size of the trial state exhibits a non-monotonic pattern. Finally we find that heterogeneity does not qualitatively affect the results.

## Results

We propose a generalized susceptible-trial-adopted-susceptible (STAS) threshold model^19^ to investigate social contagions in uncorrelated complex networks. For simplicity, we call the object that spreading as ‘behavior’, which can be used to represent ‘healthy behavior’, ‘new products’, ‘innovation’, etc. Each individual in this model is in either the susceptible, trial, or adopted state. An individual in the susceptible state may receive the information about the behavior (short as information) but has not yet reached the trail or adoption thresholds. An individual in the trial state is accepting the behavior temporarily and is willing to transmit information to susceptible neighbors. An individual in the adopted state has adopted the behavior and can transmit the information to neighbors in the susceptible and trial states.

We use the discrete updating method to renew the states of individuals^27, 28^. Initially a random fraction of *ρ*_0_ and *ξ*_0_ individuals are in the adopted and trial states, respectively, and the remaining individuals are in the susceptible state. At each time step individuals in the trial and adopted states transmit the information to neighbors in the susceptible state with probabilities *λ*_*T*_ and *λ*_*A*_, respectively. In addition, each individual in the adopted state also shares the information about social contagions with neighbors in the trial state with a probability *λ*_*AT*_. When the number of information *m* received by a susceptible individual *i* exceeds the adoption threshold *θ*_*A*_, i.e., *m* ≥ *θ*_*A*_, individual *i* enters the adoption state. When *m* is between the trial threshold *θ*_*T*_ and adoption threshold *θ*_*A*_, i.e., *θ*_*T*_ ≤ *m* < *θ*_*A*_, individual *i* enters the trial state. Similarly, when trial individual *i* in the trial state receives more than *θ*_*AT*_ number of information they enter the adopted state. Without loss of generality, we set *θ*_*A*_ ≥ *θ*_*T*_ and *θ*_*A*_ ≥ *θ*_*AT*_ because individuals in the susceptible state are more likely to enter the trial state than the adopted state, and individuals in the trial state are more like to enter the adopted state than individuals in the susceptible state. In this model of social contagions, the social reinforcement effect is included, setting it apart from simple models of contagion. Individuals in the trial and adopted states eventually lose interest in the behavior and with probability *γ* return to the susceptible state, and thus we can set *γ* = 1.0 without any loss of generality.

To describe our proposed model we use a generalized heterogeneous mean-field method^29^. In this theory, we assume that the networks have large network sizes, sparse edges, and no degree-degree correlations, and the contagion dynamics evolves continuously. Mathematically, the densities of susceptible, trial, and adopted individuals with degree *k* at time *t* are denoted *η*_*k*_(*t*), *ρ*_*k*_(*t*), and *ξ*_*k*_(*t*), respectively. At each time step each individual is either in the susceptible, trial, or adopted states. Thus the normalization condition is *η*_*k*_(*t*) + *ρ*_*k*_(*t*) + *ξ*_*k*_(*t*) = 1.

The rate at which the edges of trial individuals transmit information about social contagions to susceptible neighbors at time *t* is *ψ*_*T*_(*t*), and

1 $${\psi }_{T}(t)={{\rm{\Theta }}}_{T}(t){\lambda }_{T},$$

where Θ_*T*_(*t*) is the probability that an edge connects to a trail individual at time *t* (defined below). The rates that the edges of adopted individuals transmit the information about social contagions to susceptible *ψ*_*A*_(*t*) and trial *ψ*_*AT*_ neighbors at time *t* respectively are

2 $${\psi }_{A}(t)={{\rm{\Theta }}}_{A}(t){\lambda }_{A},$$

and

3 $${\psi }_{AT}(t)={{\rm{\Theta }}}_{A}(t){\lambda }_{AT},$$

where Θ_*A*_(*t*) is the probability that an edge connects to a adopted individual at time *t*. In uncorrelated networks the probability that an edge connects to an individual with degree *k* is *kP*(*k*)/<*k*>, where 〈*k*〉 = ∑_*k*_ *kP*(*k*) is the average degree of the network. Averaging all possible values of *k*, the expressions of Θ_*A*_(*t*) and Θ_*T*_(*t*) are

4 $${{\rm{\Theta }}}_{A}(t)=\frac{1}{\langle k\rangle }\sum _{k}kP(k){\rho }_{k}(t),$$

and

5 $${{\rm{\Theta }}}_{T}(t)=\frac{1}{\langle k\rangle }\sum _{k}kP(k){\xi }_{k}(t),$$

respectively.

During short periods of time [*t*, *t* + *dt*] susceptible individuals can obtain information (i) from trial neighbors with a probability *ψ*_*T*_(*t*)*dt* and (ii) from adopted neighbors with a probability *ψ*_*A*_(*t*)*dt*. A susceptible individual *i* with degree *k* receives *m* information from trial and adopted neighbors at time *t* with a probability

6 $${{\rm{\Omega }}}_{m}^{k}(t)=(\begin{array}{c}k\\ m\end{array}){[{\psi }_{T}(t)+{\psi }_{A}(t)]}^{m}{\mathrm{[1}-({\psi }_{T}(t)+{\psi }_{A}(t))]}^{k-m}\mathrm{.}$$

Similarly, the probability that trial individuals with degree *k* will receive *m* information from adopted neighbors at time *t* is

7 $${\varphi }_{m}^{k}(t)=(\begin{array}{c}k\\ m\end{array}){[{\psi }_{AT}(t)]}^{m}{\mathrm{[1}-{\psi }_{AT}(t)]}^{k-m}\mathrm{.}$$

Individuals in the susceptible state can enter the trial or adoption states. Susceptible individuals become adopted when the number of received information exceeds the adoption threshold *θ*_*A*_, and thus the probability of susceptible individuals that become adopted is ${\eta }_{k}(t){\sum }_{m\ge {\theta }_{A}}{{\rm{\Omega }}}_{m}^{k}(t)$. Susceptible individuals enter the trial state when their received pieces of information are equal to or exceed the trial threshold *θ*_*T*_ but are fewer than the adopted threshold *θ*_*A*_, and thus the probability that a susceptible individual will become trial is ${\eta }_{k}(t){\sum }_{m\ge {\theta }_{T}}^{m\le {\theta }_{A}-1}{{\rm{\Omega }}}_{m}^{k}(t)$. The total decrease in the density of susceptible individuals is equal to the sum of ${\eta }_{k}(t){\sum }_{m\ge {\theta }_{A}}{{\rm{\Omega }}}_{m}^{k}(t)$ + ${\eta }_{k}(t){\sum }_{m\ge {\theta }_{T}}^{m\le {\theta }_{A}-1}{{\rm{\Omega }}}_{m}^{k}(t)$. The increase in the density of susceptible individuals occurs (i) when adopted individuals lose interest in the social contagions and revert to the susceptible state [the fraction of adopted individuals with degree *k* reverting to the susceptible state is *ρ*_*k*_(*t*)] and (ii) when individuals in the trial state receiving fewer number of information than the threshold *θ*_*AT*_ revert to the susceptible state [the fraction of trial individuals reverting to the susceptible state is ${\xi }_{k}(t){\sum }_{m < {\theta }_{AT}(t)}{\varphi }_{m}^{k}(t)$]. The time evolution of the fraction of the degree *k* susceptible individuals is given by

8 $$\begin{array}{rcl}\frac{d{\eta }_{k}(t)}{dt} & = & {\rho }_{k}(t)+{\xi }_{k}(t)\sum _{m < {\theta }_{AT}(t)}{\varphi }_{m}^{k}(t)-{\eta }_{k}(t)[\sum _{m\ge {\theta }_{A}}{{\rm{\Omega }}}_{m}^{k}(t)+\sum _{m\le {\theta }_{T}}^{{\theta }_{A}-1}{{\rm{\Omega }}}_{m}^{k}(t)]\\  & = & {\rho }_{k}(t)+{\xi }_{k}(t)\sum _{m < {\theta }_{AT}}{\varphi }_{m}^{k}(t)-{\eta }_{k}(t)\sum _{m\ge {\theta }_{T}}{{\rm{\Omega }}}_{m}^{k}(t),\end{array}$$

where ${\eta }_{k}(t){\sum }_{m\ge {\theta }_{T}}{{\rm{\Omega }}}_{m}^{k}(t)$ is the probability of susceptible individuals with degree *k* entering the trial or adopted states.

Similar to the evolution of the density of susceptible individuals, the increasing of *ρ*_*k*_(*t*) equals the sum of the fraction of trial individuals who received information equal to or greater than *θ*_*AT*_ and enter the adopted state, and the fraction of susceptible individuals who received information equal to or greater than *θ*_*A*_ and enter the adopted state. The fraction of trial individuals with degree *k* entering the adopted state is ${\xi }_{k}(t){\sum }_{m\ge {\theta }_{AT}}{\varphi }_{m}^{k}(t)$. The increasing density of susceptible individuals entering the adopted state is equal to the decreasing density of adopted individual reverting to the susceptible state, i.e., ${\eta }_{k}(t){\sum }_{m\ge {\theta }_{A}}{{\rm{\Omega }}}_{m}^{k}(t)$. The decreasing density of adopted individuals with degree *k* equals the fraction of the adopted reverting to the susceptible state, i.e., *ρ*_*k*_(*t*). Analogously, we can derive the time evolution of adopted individuals with degree *k* as

9 $$\frac{d{\rho }_{k}(t)}{dt}={\xi }_{k}(t)\sum _{m\ge {\theta }_{AT}}{\varphi }_{m}^{k}(t)+{\eta }_{k}(t)\sum _{m\ge {\theta }_{A}}{{\rm{\Omega }}}_{m}^{k}(t)-{\rho }_{k}(t\mathrm{).}$$

In addition, the density of trial individuals increases when susceptible individuals move to the trial state when the number of information they received is equal to or greater than the trial threshold but fewer than the adopted threshold, i.e., *θ*_*T*_ ≤ *m* ≤ *θ*_*A*_ − 1. As stated above, some of the trial individuals move to the adopted state with a probability ${\xi }_{k}(t){\sum }_{m\ge {\theta }_{AT}(t)}{\varphi }_{m}^{k}(t)$ when the number of information they received is equal to or greater than *θ*_*AT*_. The remaining trial individuals who receive fewer than *θ*_*AT*_ revert to the susceptible state, i.e., ${\xi }_{k}(t){\sum }_{m < {\theta }_{AT}(t)}{\varphi }_{m}^{k}(t)$. Thus the decreasing density of trial individuals equals the sum of the above, and we can derive the time evolution equation of the trial individuals with degree *k* as

10 $$\begin{array}{rcl}\frac{d{\xi }_{k}(t)}{dt} & = & {\eta }_{k}(t)\sum _{m\ge {\theta }_{T}}^{{\theta }_{A}-1}{{\rm{\Omega }}}_{m}^{k}(t)-{\xi }_{k}(t)\sum _{m\ge {\theta }_{AT}(t)}{\varphi }_{m}^{k}(t)-{\xi }_{k}(t)\sum _{m < {\theta }_{AT}(t)}{\varphi }_{m}^{k}(t)\\  & = & {\eta }_{k}(t)\sum _{m\ge {\theta }_{T}}^{{\theta }_{A}-1}{{\rm{\Omega }}}_{m}^{k}(t)-{\xi }_{k}(t).\end{array}$$

Equations (8)–(10) describe the evolution of the model. We find the density of each type of individual *X* ∈ {*η*, *ρ*, *ξ*} at a given time *t* to be

11 $$X(t)=\sum _{k}P(k){X}_{k}(t),$$

where *P*(*k*) is the degree distribution of the network. When *t* → ∞, we have *dη*_*k*_(*t*)/*dt* = 0, *dρ*_*k*_(*t*)/*dt* = 0 and *dξ*_*k*_(*t*)/*dt* = 0. For simplicity, we denote *η*_*k*_(∞), *ρ*_*k*_(∞), *ξ*_*k*_(∞), ${{\rm{\Omega }}}_{m}^{k}(\infty )$ and ${\varphi }_{m}^{k}(\infty )$ as *η*_*k*_, *ρ*_*k*_, *ξ*_*k*_, ${{\rm{\Omega }}}_{m}^{k}$ and ${\varphi }_{m}^{k}$, respectively. As *η*_*k*_ = 1 − *ρ*_*k*_ − *ξ*_*k*_, Eqs (8) and (9) can be written as

12 $$\begin{array}{rcl}{\rho }_{k} & = & (1-{\rho }_{k}-{\xi }_{k})\sum _{m\ge {\theta }_{T}}{{\rm{\Omega }}}_{m}^{k}-{\xi }_{k}\sum _{m < {\theta }_{AT}}{\varphi }_{m}^{k}\\  & = & {g}_{1}({\rho }_{k},{\xi }_{k})\end{array}$$

and

13 $$\begin{array}{rcl}{\rho }_{k} & = & {\xi }_{k}\sum _{m\ge {\theta }_{AT}}{\varphi }_{m}^{k}+(1-{\rho }_{k}-{\xi }_{k})\sum _{m\ge {\theta }_{A}}{{\rm{\Omega }}}_{m}^{k}\\  & = & {g}_{2}({\rho }_{k},{\xi }_{k}),\end{array}$$

respectively. Numerically solving Eqs (12)–(13), we obtain the fraction of individuals in the adopted and trial states as *ρ* = ∑_*k*_ *P*(*k*)*ρ*_*k*_ and *ξ* = ∑_*k*_ *P*(*k*)*ξ*_*k*_, respectively. Another important issue is the critical point that determines the probability of an outbreak of social contagions. See details in the Method Section.

### Numerical simulations

We now perform extensive simulations of the STAS model on random-regular (RR), Erdös-Rényi (ER), and scale-free (SF) networks. We set the network size, recovery probability, and adoption threshold to be *N* = 5,000, *γ* = 1.0, and *θ*_*A*_ = 4, respectively. For RR and ER networks, we set the average degree 〈*k*〉 = 10. For other values of parameters, we perform extensive numerical simulations in the Supporting Information, and find that the results are not qualitatively affected. The dynamics terminate when there is no individuals in the adopted and trail states, or the time steps reach a give value *t*_max_ = 1,000. We carry out at least 1000 independent dynamical realizations and calculate the average values.

### Random-Regular networks

**Figure 1:**
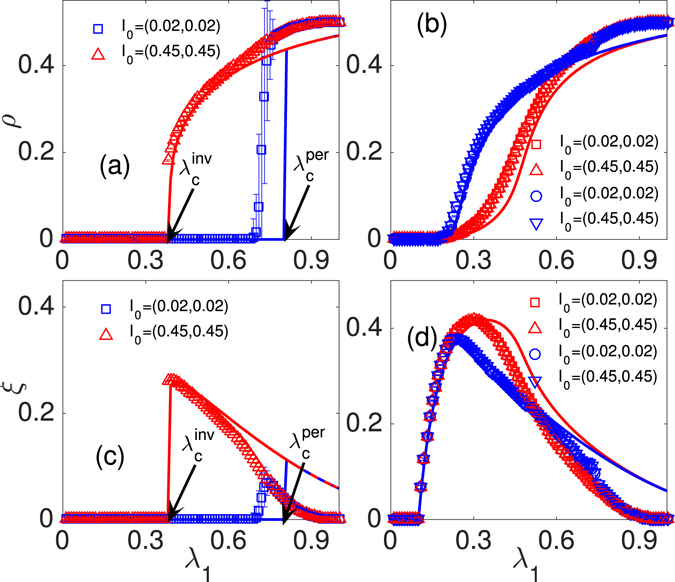
The final fraction of individuals in the adopted and trail state versus the information sharing probability *λ* on RR network. The final fraction of individuals in the adopted state *ρ* [(**a**) and (**b**)] and trial state *ξ* [(**c**) and (**d**)] versus *λ*_1_ at ${I}_{0}=(0.02,0.02)$ (◽, ⚪) and ${I}_{0}=(0.45,0.45)$ (∆, ∇). We set $\mathop{\theta }\limits^{\longrightarrow}=\mathrm{(4,\; 2,\; 1)}$ in (**a**) and (**c**), $\mathop{\theta }\limits^{\longrightarrow}=(4,1,2)$ for the red ◽ and ∆ in (**b**) and (**d**), and $\mathop{\theta }\limits^{\longrightarrow}=(4,1,1)$ for the blue ⚪ and ∇ in (**b**) and (**d**). Symbols represent simulation results and lines are theoretical predictions.

Figure 1 shows social contagions on RR networks with different threshold values $\mathop{\theta }\limits^{\longrightarrow}=({\theta }_{A},{\theta }_{T},{\theta }_{AT})$. Figure 1(a,c) show $\mathop{\theta }\limits^{\longrightarrow}\mathrm{=(4,2,1)}$. For a small fraction of seeds, i.e., ${I}_{0}=({\rho }_{0},{\xi }_{0})=$ $(0.02,0.02)$, *ρ* increases discontinuously with *λ*_1_ = *λ*_*A*_ = *λ*_*T*_ = *λ*_*AT*_ because a finite fraction of individuals adopt the behavior simultaneously. The presence threshold ${\lambda }_{c}^{{\rm{per}}}$ ^30, 31^ separates the system into absorbing and active regions. Similar to disease spreading dynamics^29^, a vanishingly small fraction of individuals adopt the behavior in the absorbing region, and a finite fraction of individuals adopted the behavior in the active region. Note that *ξ* first increases discontinuously to a peak when *λ*_1_ is larger than the presence threshold ${\lambda }_{c}^{{\rm{pre}}}$, and then decreases continuously with *λ*_1_. The discontinuously increasing of *ρ* is induced by a finite fraction of susceptible individuals moving to the trial state when the value of *λ*_1_ is small. For a large *λ*_1_, susceptible individuals receive more information to become adopted, and the probability that trail individuals will become adopted is larger. Thus *ξ* continuously decreases and *ρ* increases. For a large fraction of seeds, i.e., ${I}_{0}=(0.45,0.45)$, we find similar phenomena for *ρ* and *ξ*. Here the critical point is an invasion threshold ${\lambda }_{c}^{{\rm{inv}}}$ ^30, 31^.

The above phenomena indicate that the system contains hysteresis loop of our proposed mathematical model in both simulations and theory. Specifically, the fraction of adopted (trial) individuals versus *λ*_1_ depends on the initial conditions of ${I}_{0}=({\rho }_{0},{\xi }_{0})$ at region ${\lambda }_{c}^{{\rm{inv}}} < {\lambda }_{1} < {\lambda }_{c}^{{\rm{per}}}$. In this region there are no individuals in the adopted (trial) state for a small fraction of seeds [e.g., ${I}_{0}=(0.02,0.02)$], and a finite fraction of individuals in the adopted (trial) state for a large fraction of seeds [e.g., ${I}_{0}=(0.45,0.45)$]. The hysteresis phenomena in this social contagions are induced by social reinforcement. We find that the hysteresis phenomena disappears once susceptible individuals are more likely to enter the trial state [see Fig. 1(b,d)]. Our theory agrees well with the simulation results in most cases, and differences between theory and simulation are induced by the strong dynamical correlations among the states of neighbors and finite-size network^5^.

**Figure 2:**
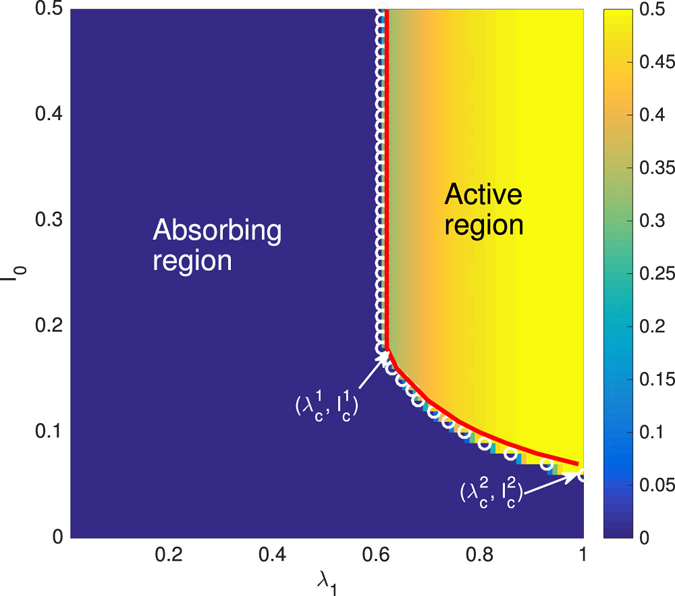
Color-coded of the final adoption size *ρ* from simulations versus the initial seeds *I*_0_ and information transmission probability *λ*_1_ with $\mathop{\theta }\limits^{\longrightarrow}=(4,3,1)$. The critical points *λ*_*c*_ separate the (*λ*_1_, *I*_0_) into absorbing and active regions. The symbols and lines are the numerical and theoretical predictions of *λ*_*c*_, respectively.

We next investigate the effect of the initial condition (i.e., *ρ*_0_ = *ξ*_0_ = *I*_0_) and the information transmission probability on the spreading dynamics on RR networks with $\mathop{\theta }\limits^{\longrightarrow}=(4,3,1)$. Figure 2 shows the values of *ρ* in the *I*_0_ − *λ*_1_ plane. We find that *ρ* increases with *λ*_1_, since individuals in the network have more opportunities to get the information. Based on the values of *ρ*, the phase diagram is divided into an absorbing region (i.e., a local behavior adoption region) and an active region (i.e., a global behavior adoption region) by the critical points *λ*_*c*_. The red line and white circles represent the theoretical and numerical critical points, respectively. We find a critical fraction of seeds, denoted ${I}_{c}^{2}$, below which no information transmission probability *λ*_1_ can trigger a global behavior adoption since susceptible individuals have little opportunity to receive the information. When ${I}_{0} > {I}_{c}^{2}$, global behavior adoption becomes possible. When ${I}_{c}^{2} < {I}_{0}\le {I}_{c}^{1}$, the seed size markedly affects the final adoption size *ρ* and the critical points *λ*_*c*_. Specifically, the value of *ρ* (*λ*_*c*_) increases (decreases) with *I*_0_ because individuals have more opportunities to receive the information, i.e., the system has a hysteresis loop in this region. When *I*_0_ is large, i.e., when ${I}_{0} > {I}_{c}^{1}$, the seed size does not affect *ρ* and *λ*_*c*_, since few susceptible received the information and the system becomes saturated.

**Figure 3:**
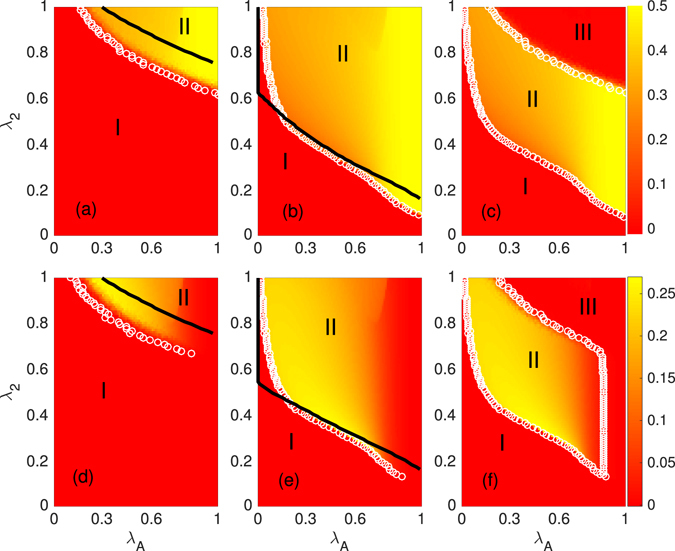
Color-coded simulation results of the final adoption size *ρ* (**a**–**c**) and trial size *ξ* (**d**–**f**) versus *λ*_*A*_ and *λ*_2_ with $\mathop{\theta }\limits^{\longrightarrow}=(4,2,1)$. The seed sizes of (**a**) and (**d**) are set as ${I}_{0}=(0.02,0.02)$, and the seed sizes in (**b**) and (**e**) are ${I}_{0}=(0.45,0.45)$. (**c**) The difference between the values of *ρ* in (**a**) and (**b**). (**f**) The difference between the values of *ξ* in (**d**) and (**e**). The critical points divide the *λ*_*A*_ − *λ*_2_ plane into different regions. The symbols and lines are the numerical and theoretical predictions of *λ*_*c*_, respectively.

We next determine the final numbers of trial and adopted individuals as a function of the transmission probabilities *λ*_*A*_ and *λ*_2_ = *λ*_*T*_ = *λ*_*AT*_, with $\overrightarrow{\theta }=(4,2,1)$, as shown in Fig. 3. We find that *ρ* increases with *λ*_*A*_ and *λ*_2_ since both susceptible and trial individuals have a larger probability of adopting the social contagions [see Fig. 3(a,b)]. However, *ξ* first increases with *λ*_*A*_ and then decreases for a given *λ*_2_ [see Fig. 3(d,e)]. Initially susceptible individuals have a larger probability of receiving enough information about social contagions to enter trial status when *λ*_*A*_ increases, and at first *ξ* increases with *λ*_*A*_. However, when *λ*_*A*_ is very large, susceptible individuals receive sufficient information to reach adoption threshold *θ*_*A*_, and the probability that they will enter the trial state decreases, and thus *ξ* decreases. Based on the final size values, the phase diagram is divided into two regions having different critical transmission probabilities [see Fig. 3(a,b,d,e)]. Specifically, the presence threshold (invasion threshold) divides the plane into an absorbing region (i.e., region I) and an active region (i.e., region II) for a small (large) value of seeds, and because individuals now have a higher probability of receiving the information, the two thresholds decrease with *λ*_*A*_. Note that the invasion threshold values are different for different seed sizes. Thus we see the hysteresis loop [region II of Fig. 3(c,f)]. In region II of Fig. 3(c,f), the different seed sizes strongly affect the final trial and adoption sizes.

### Heterogeneous networks

**Figure 4:**
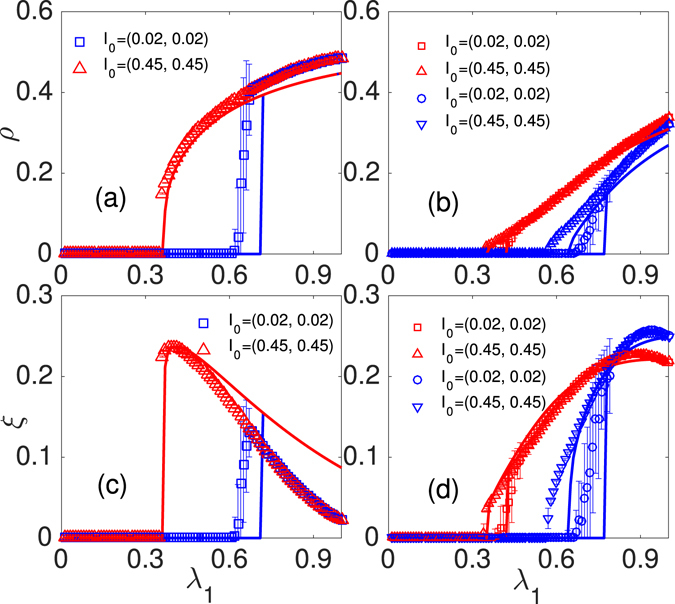
The numerical simulation results of the final adoption size *ρ* (**a**,**b**) and trial size *ξ* (**c**,**d**) versus different values of *λ*_1_ with $\mathop{\theta }\limits^{\longrightarrow}=(4,\,2,\,1)$, ${I}_{0}=(0.02,0.02)$ (◽, ○) and ${I}_{0}=(0.45,0.45)$ (Δ, ∇). In (**a**) and (**c**), social contagions on ER networks. In (**b**) and (**d**), social contagions on SF networks with *τ* = 2.5 (◽ and Δ) and *τ* = 3.0 (○ and ∇).

Figure 4 shows how differing network topological parameters affect the dynamics of social contagions. Figure 4(a,c) show hysteresis phenomena in ER networks. The average degree of the ER networks in our simulation is 〈*k*〉 = 10. Similar to the phenomena in RR networks, in a small fraction of seeds there are no adopted (trial) individuals [e.g., ${I}_{0}=(0.02,0.02)$], *ρ* increases discontinuously with *λ*_*c*_, and *ξ* increases discontinuously and then decreases continuously with increasing *λ*_1_.

Figure 4(b,d) show the how scale-free (SF) networks affect the social contagions. SF networks are generated using the uncorrelated configuration model^32, 33^ with a power-law degree distribution $P(k) \sim {k}^{-\tau }$, where *τ* is the degree exponent, *k*_min_ = 3 and ${k}_{{\rm{\max }}}=\sqrt{N}$ are the minimum and maximum degrees, respectively. We find that network topology does not qualitatively affect hysteresis loop phenomena and the growth patterns of the trial and adopted individuals in RR networks. Figure 4(b,d) show that *ρ* and *ξ* increase continuously at both ${I}_{0}=(0.02,0.02)$ and ${I}_{0}=(0.45,0.45)$ in both degree exponent *τ* = 2.5 and *τ* = 3.0. We find that because there are more hubs in heterogeneous networks, increasing the heterogeneity of the degree distribution (i.e., by using smaller values of the degree exponent) reduces the value of *λ*_*c*_. Our suggested theory can qualitatively describe the about phenomena, the deviations between theoretical predictions and simulation results derive from the strong dynamical correlations among the states of neighbors^5^.

## Discussion

We have proposed a novel susceptible-trial-adopted-susceptible (STAS) model for describing the social contagions in complex networks. We use it to examine how individuals can enter a trial period prior to adopting social contagion. Based on extensive numerical simulations and generalized heterogeneous mean-field theory, we find a hysteresis loop in our particular mathematical model of social contagions, i.e., the initial conditions of the dynamics affect the final state of the dynamics. In addition, the number of individuals adopting the behavior increases discontinuously with the transmission probability of the information, but the number of individuals who enter trial status first increases discontinuously then decreases continuously. Finally, we find that network topology does not qualitatively affect the results. These results provide us with a deeper understanding of how social contagions occur on complex networks, and they enrich our knowledge of phase transitions^10, 34^. Furthermore, the verification of our results in real-data is an interesting research topic.

## Methods

### Determine the critical point

Another important issue is the critical point that determines the probability of an outbreak of social contagions. For the case of random-regular networks, we obtain the critical condition^35, 36^

14 $$\frac{\partial F(\rho ,\xi )}{\partial \xi }\frac{\partial G(\rho ,\xi )}{\partial \rho }=\frac{\partial F(\rho ,\xi )}{\partial \rho }\frac{\partial G(\rho ,\xi )}{\partial \xi },$$

where *F*(*ρ*, *ξ*) = *g*_1_(*ρ*, *ξ*) − *ρ* and *G*(*ρ*, *ξ*) = *g*_2_(*ρ*, *ξ*) − *ρ*. Numerically solving Eqs (12)–(13) and (14), we find the critical transmission probabilities ${\overrightarrow{\lambda }}_{c}=({\lambda }_{A}^{c},{\lambda }_{T}^{c},{\lambda }_{AT}^{c})$.

## Electronic supplementary material

Supporting Information
