# Supplementary material for: Emergence of hysteresis loop in social contagions on complex networks
Source: Sci Rep. 2017 Jul 21;7:6103. doi: 10.1038/s41598-017-06286-w (PMC5522431; doi:10.1038/s41598-017-06286-w)
Supplement: Supplementary file 1 — Supporting Information [file 41598_2017_6286_MOESM1_ESM.pdf]

Supporting information of 'Emergence of hysteresis loop in social contagions on complex networks'

Zhen Su,[1,2] Wei Wang,[3,4,2,*] Lixiang Li,[5,†] Jinghua Xiao,[1] and H. Eugene Stanley[2]

[1]*School of Science, Beijing University of Posts and Telecommunications, Beijing 100876, China*
[2]*Center for Polymer Studies and Department of Physics, Boston University, Boston, Massachusetts 02215, USA*
[3]*Web Sciences Center, University of Electronic Science and Technology of China, Chengdu 610054, China*
[4]*Big Data Research Center, University of Electronic Science and Technology of China, Chengdu 610054, China*
[5]*Information Security Center, State Key Laboratory of Networking and Switching Technology, Beijing University of Posts and Telecommunications, Beijing 100876, China*

(Dated: June 6, 2017)

* wwzqbx@hotmail.com
† li_lixiang2006@163.com

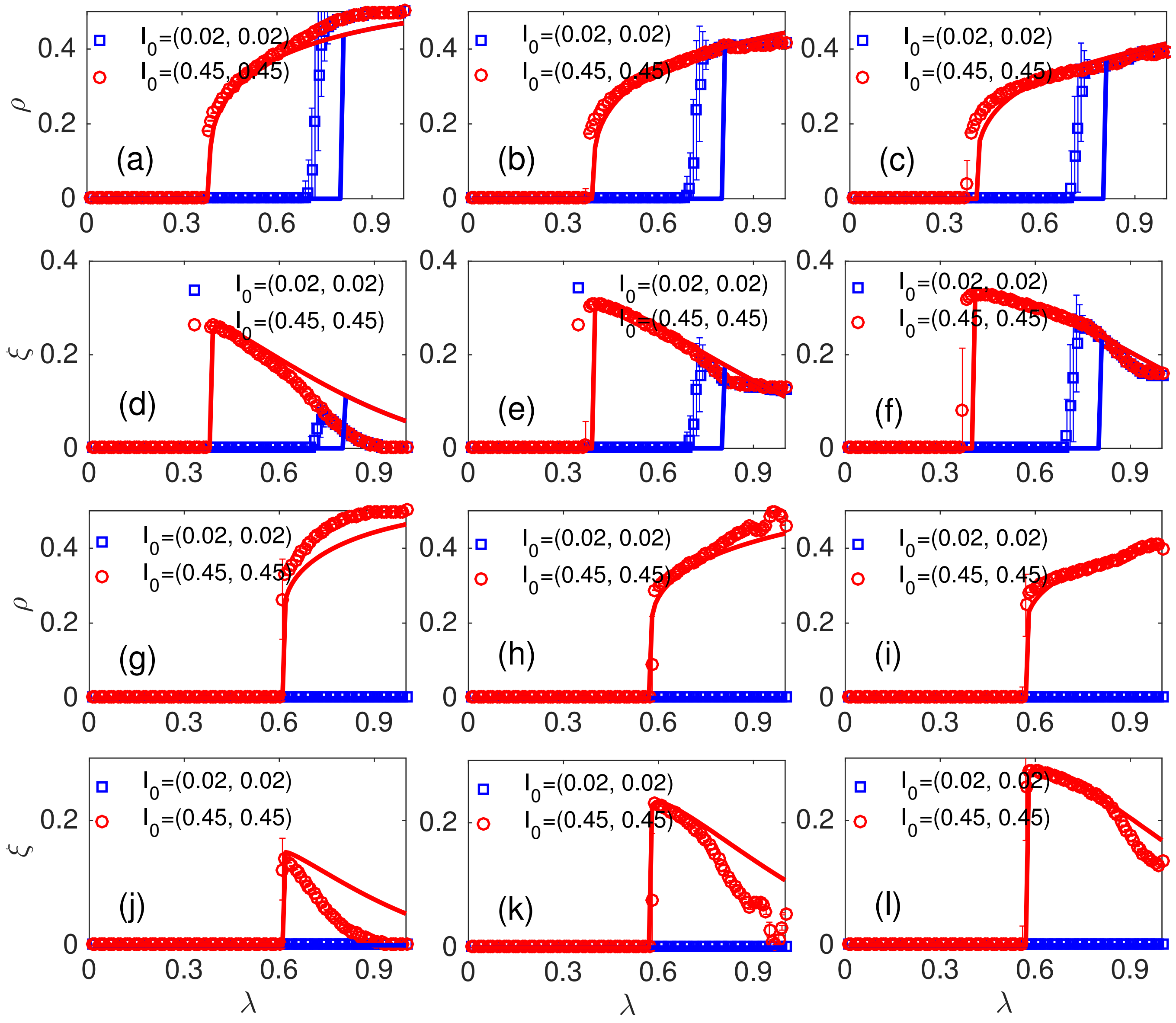

FIG. 1. (Color online) **Social contagions on RR networks.** The final fraction of individuals in the adopted state ρ (a)-(c) and (g)-(i), and trial state ξ (d)-(f) and (j)-(l) versus λ_1 at $\overrightarrow{I_0} = (0.02, 0.02)$ (□) and $\overrightarrow{I_0} = (0.45, 0.45)$ (◯). We set $\overrightarrow{\theta} = (4, 2, 1)$ in (a) and (d), $\overrightarrow{\theta} = (5, 2, 1)$ in (b) and (e), $\overrightarrow{\theta} = (6, 2, 1)$ in (c) and (f), $\overrightarrow{\theta} = (4, 3, 1)$ in (g) and (j), $\overrightarrow{\theta} = (5, 3, 1)$ in (h) and (k) and $\overrightarrow{\theta} = (6, 3, 1)$ in (i) and (l). Symbols represent simulation results and lines are theoretical predictions.

We investigate the effect of the initial condition $\overrightarrow{I}_0$ and the information transmission probability on the spreading dynamics of RR, ER and SF networks with different values of $\overrightarrow{\theta}$.

On RR and ER networks, as shown respectively in Figs. 1 and 2, we find that the hysteresis loop does not affected by the values of $\overrightarrow{\theta}$. The suggested mean-field theory agree well with the numerical simulations, the deviations between theoretical predictions and simulation results derive from the strong dynamical correlations among the states of neighbors.

On SF networks with different degree exponents as shown in Figs. 3, we find that the hysteresis loop does not affected by the values of $\overrightarrow{\theta}$.

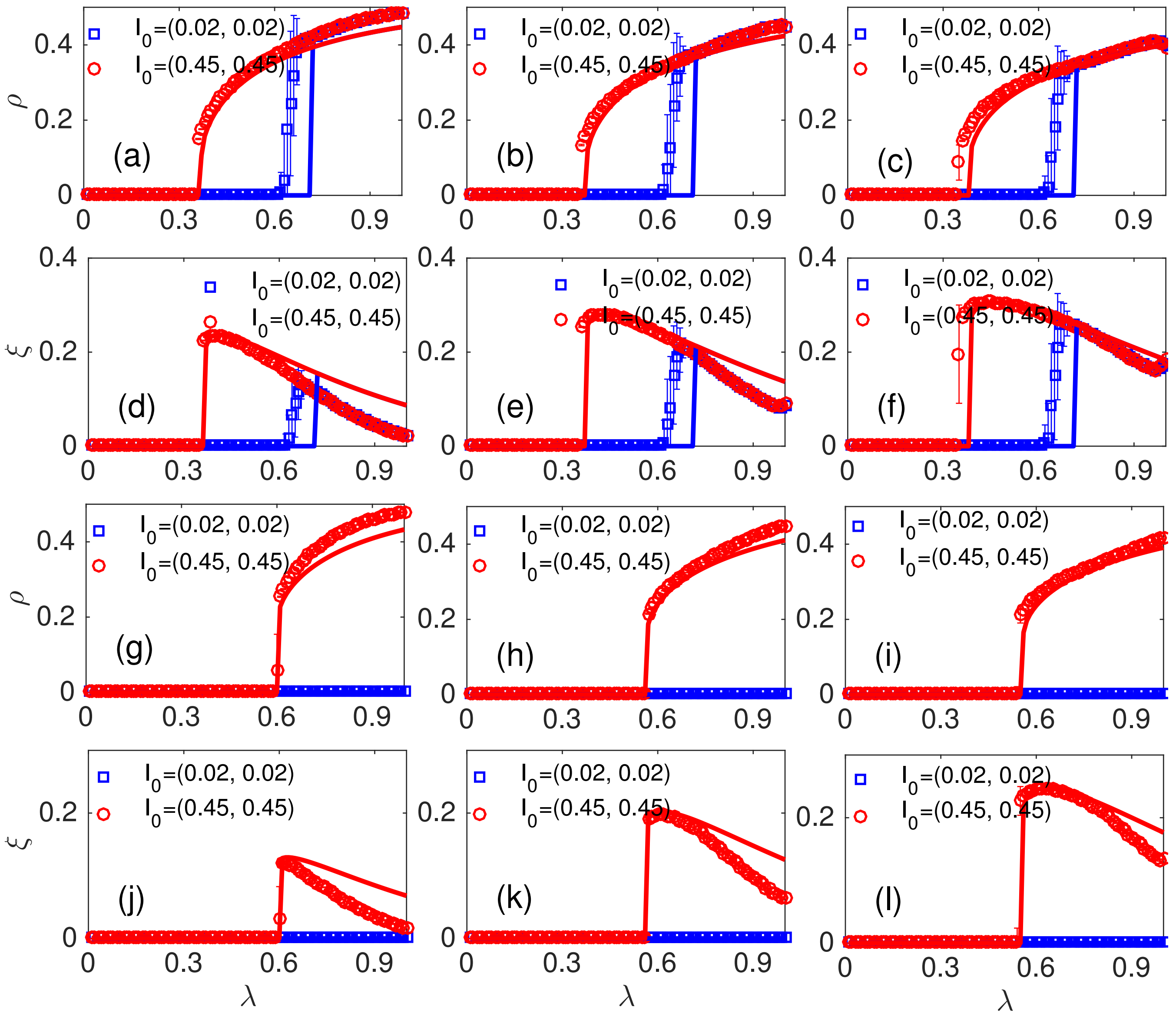

FIG. 2. (Color online) **Social contagions on ER networks.** The final fraction of individuals in the adopted state ρ (a)-(c) and (g)-(i), and trial state ξ (d)-(f) and (j)-(l) versus λ_1 at $\overrightarrow{I_0} = (0.02, 0.02)$ (□) and $\overrightarrow{I_0} = (0.45, 0.45)$ (◯). We set $\overrightarrow{\theta} = (4, 2, 1)$ in (a) and (d), $\overrightarrow{\theta} = (5, 2, 1)$ in (b) and (e), $\overrightarrow{\theta} = (6, 2, 1)$ in (c) and (f), $\overrightarrow{\theta} = (4, 3, 1)$ in (g) and (j), $\overrightarrow{\theta} = (5, 3, 1)$ in (h) and (k) and $\overrightarrow{\theta} = (6, 3, 1)$ in (i) and (l). Symbols represent simulation results and lines are theoretical predictions.

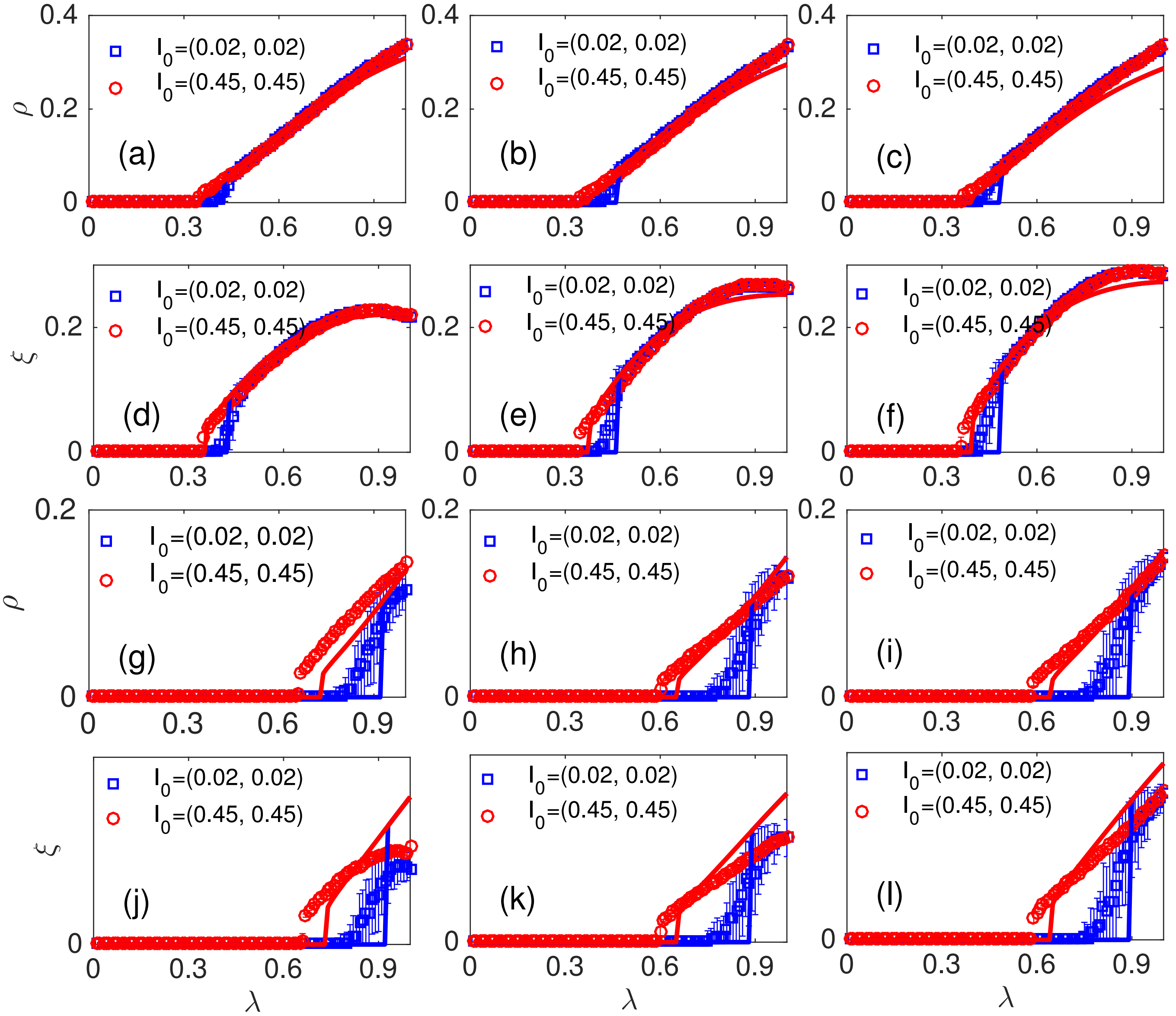

FIG. 3. (Color online) **Social contagions on SF networks with** $\tau = 2.5$**.** The final fraction of individuals in the adopted state ρ (a)-(c) and (g)-(i), and trial state ξ (d)-(f) and (j)-(l) versus λ_1 at $\overrightarrow{I_0} = (0.02, 0.02)$ (□) and $\overrightarrow{I_0} = (0.45, 0.45)$ (◯). We set $\overrightarrow{\theta} = (4, 2, 1)$ in (a) and (d), $\overrightarrow{\theta} = (5, 2, 1)$ in (b) and (e), $\overrightarrow{\theta} = (6, 2, 1)$ in (c) and (f), $\overrightarrow{\theta} = (4, 3, 1)$ in (g) and (j), $\overrightarrow{\theta} = (5, 3, 1)$ in (h) and (k) and $\overrightarrow{\theta} = (6, 3, 1)$ in (i) and (l). Symbols represent simulation results and lines are theoretical predictions.
